# Interplay between Superoxide Dismutase, Glutathione Peroxidase, and Peroxisome Proliferator Activated Receptor Gamma Polymorphisms on the Risk of End-Stage Renal Disease among Han Chinese Patients

**DOI:** 10.1155/2016/8516748

**Published:** 2016-01-06

**Authors:** Chia-Ter Chao, Yen-Ching Chen, Chih-Kang Chiang, Jenq-Wen Huang, Cheng-Chung Fang, Chen-Chih Chang, Chung-Jen Yen

**Affiliations:** ^1^Department of Medicine, National Taiwan University Hospital Jinshan Branch, New Taipei City, Taiwan; ^2^Graduate Institute of Toxicology, College of Medicine, National Taiwan University, No. 1 Jen-Ai Road, Section 1, 10051 Taipei, Taiwan; ^3^Institute of Epidemiology and Preventive Medicine, College of Public Health, National Taiwan University, No. 17 Xu-Zhou Road, 10055 Taipei, Taiwan; ^4^Department of Integrated Diagnostics & Therapeutics, National Taiwan University Hospital, No. 7 Chung-Shan South Road, 10002 Taipei, Taiwan; ^5^Department of Internal Medicine, National Taiwan University Hospital, No. 7 Chung-Shan South Road, 10002 Taipei, Taiwan; ^6^Department of Emergency Medicine, National Taiwan University Hospital, No. 7 Chung-Shan South Road, 10002 Taipei, Taiwan; ^7^Department of Geriatric Medicine and Gerontology, National Taiwan University Hospital, No. 7 Chung-Shan South Road, 10002 Taipei, Taiwan

## Abstract

*Background*. Single nucleotide polymorphisms (SNPs) of antioxidants, including superoxide dismutase 2 (SOD2) and glutathione peroxidase 1 (GPX1), play an important role in the risk for cancer and metabolic disorders. However, little is known regarding the effect of antioxidant SNPs on renal events.* Methods*. We prospectively enrolled multicenter patients with end-stage renal disease (ESRD) and those without chronic kidney disease (CKD) of Han Chinese origin, with SOD2 (Val16Ala), GPX1 (Pro197Leu), and PPAR-*γ* (Pro12Ala, C161T) genotyped. Multiple regression analyses were conducted to evaluate the significant risk determinants for ESRD.* Results*. Compared to ESRD patients, non-CKD subjects were more likely to have T allele at SOD2 Val16Ala (*p* = 0.036) and CC genotype at PPAR-*γ* Pro12Ala (*p* = 0.028). Regression analysis showed that TT genotype of SOD2 Val16Ala conferred significantly lower ESRD risk among patients without diabetes (odds ratio 0.699; *p* = 0.018). GPX1 SNP alone did not alter the risk. We detected significant interactions between SNPs including PPAR-*γ* Pro12Ala, C161T, and GPX1 regarding the risk of ESRD.* Conclusion*. This is the first and largest study on the association between adverse renal outcomes and antioxidant SNPs among Han Chinese population. Determination of SOD2 and PPAR-*γ* SNPs status might assist in ESRD risk estimation.

## 1. Introduction

Patients with persistent renal failure are increasing worldwide, and the number is expected to rise even higher as the population age with multimorbidity. This creates tremendous burden on the existent healthcare resources. In USA, the annual expenditure for the care of end-stage renal disease (ESRD) patients reaches nearly 50 billion dollars in 2011, with an incremental range of 2.8 to 3.4% per year [1]. Given this unavoidable trend, researches focusing on the risk factors and mortality determinants for ESRD assume increasing importance.

Oxidative stress is proposed to play an important role in the pathogenesis of diseases including cardiometabolic diseases, neurodegenerative diseases, neoplasm formation, and, most important of all, chronic kidney disease (CKD) [2–4]. Surge of oxidative stress is frequently linked to reactive oxygen species (ROS) formation and/or lowering antioxidant capacity, incurring damage to the surrounding tissues and organ injury. Defending against oxidative stress, antioxidants could be categorized as exogenous (carotene, tocopherol, ascorbate, etc.) and endogenous, especially thiol group-containing macromolecules and enzymes [5]. There are reports suggesting that prooxidant levels increase and antioxidant enzyme activities decrease stepwise as renal function deteriorates [6]. Moreover, ROS accumulation could not only perpetuate immunogenic glomerular/interstitial injury, but also contribute to endothelial dysfunction over the long run, further deranging the intraglomerular and systemic hemodynamics [7].

Among the endogenous antioxidants, superoxide dismutase (SOD) and glutathione peroxidase (GPX) are particularly important players. Several studies have investigated the risk of CKD conferred by the genetic heterogeneity of these two genes. Both SOD1 and SOD2 single nucleotide polymorphisms (SNPs) are found to be associated with 2- to 3-fold higher risk of developing diabetic nephropathy in type 1 and 2 diabetes mellitus (DM) patients [8, 9]. CKD patients carrying SOD2 Ala16Val genotype also had greater decline of estimated glomerular filtration rate (eGFR) during follow-up [10]. Similarly, plasma GPX activity also correlated with annual eGFR change in CKD patients [10]. However, very few studies focused on Asian population, and none of the past reports addressed the influence of SNPs of these antioxidants, especially SOD and GPX, on the risk of dialysis-dependent ESRD patients. Previously, we have identified that peroxisome proliferator activated receptor- (PPAR-) *γ* SNPs, Pro12Ala, and C161T significantly modified the risk of developing ESRD and prognosis of ESRD patients of Han origin [11]. In the current study, we aimed to determine the relationship between SOD2, GPX1, PPAR-*γ* genotypes, and the risk of developing ESRD among patients of Han origin, using a large multicenter cohort.

## 2. Material and Methods

### 2.1. Study Population

In this multicenter study, we identified ESRD patients (*n* = 671) who initiated long-term hemodialysis or peritoneal dialysis from 3 hospitals and 9 clinics in the northern Taiwan between 2002 and 2003 [12–15]. Another 780 patients without chronic kidney disease (CKD) were recruited from the health checkup services during the same time period. Blood samples were collected from each subject at the time of enrollment, and individuals were prospectively followed up. The current study was approved by the institutional review boards of all participating institutions.

All subjects completed a questionnaire about their sociodemographic status (e.g., birthday, sex, and education), comorbidity history (e.g., hypertension and diabetes), and lifestyle factors (e.g., smoking and alcohol consumption) with the assistance of general practitioners. Diabetes was determined based on a history of physician-diagnosed diabetes or 2 separate episodes of fasting blood glucose levels ≥126 mg/dL (7.0 mmole/L). The causes of ESRD and the vintage were recorded for all ESRD cases at enrollment. Diabetic nephropathy as the cause of ESRD was recognized if the patient had a long-standing diabetes history (at least 5 years) before dialysis start, presence of diabetic retinopathy, and/or proteinuria (≥100 mg/dL by urinalysis) without other causes.

### 2.2. Laboratory Assays (Genotyping)

Genomic DNA was extracted from peripheral blood leukocytes using the Chemagic DNA Blood kits (Chemagen AG, Baesweiler, Germany). Genotypes of the SNPs were determined by the polymerase chain reaction-restriction fragment length polymorphism (PCR-RFLP). Fragments encompassing the SNPs (V16A of SOD2, rs1050450 of GPX1, P12A, and C161T of PPAR-*γ*) were generated from genomic DNA by PCR using the primer pairs shown in Table 1. The PCR products were then digested with restriction enzymes (New England BioLabs, Beverly, MA, USA), electrophoresed on a 2.5% agarose gel, and stained with ethidium bromide. The size of the digested PCR products for each SNP variant was also summarized in Table 1.

### 2.3. Statistical Analysis

Data analysis was performed using the SAS, 9.1.3 software (SAS Institute Inc., Cary, NC, USA) and the R, 2.11.1 software (R Foundation for Statistical Computing, Vienna, Austria). In statistical testing, two-sided *p* value ≤ 0.05 was considered statistically significant. The distributional properties of continuous variables were expressed as mean ± standard deviation (SD), whereas categorical variables were presented as frequency and percentage.

The potential risk factors for ESRD were examined in univariate analysis using chi-square test, Fisher's exact test, two-sample *t*-test, Wilcoxon rank-sum test, and log-rank test, as appropriate. Multivariate analysis was subsequently conducted using weighted logistic regression model to estimate the effects of risk factors on the probability of long-term dialysis.

To ensure the quality of analysis results, basic model-fitting techniques for (1) variable selection, (2) goodness-of-fit (GOF) assessment, and (3) regression diagnostics were used in our regression analyses. Specifically, the stepwise variable selection procedure (with iterations between the forward and backward steps) was applied to obtain the candidate final regression model. All the univariate significant and nonsignificant relevant covariates listed in Table 3 and their interaction variables were selected and the significance levels for entry (SLE) and for stay (SLS) were set to 0.15 or larger (up to 0.35). The best final regression model was identified by reducing the significance levels to 0.05. Both the GOF measures, including the percentage of concordant pairs, estimated area under the receiver operating characteristic (ROC) curve, and adjusted generalized *R* ^2^, and the GOF tests, including the deviance GOF test, Pearson chi-squared GOF test, and Hosmer-Lemeshow GOF test (for logistic regression model), and, if feasible, Grønnesby-Borgan GOF test (for Cox's proportional hazards model), were examined to assess the GOF of the fitted regression models. The generalized additive models (GAM) were applied to detect nonlinear effects of continuous covariates.

## 3. Results

### 3.1. Clinical Features of Enrollees

The recruited numbers of ESRD patients and their non-CKD counterparts are comparable. Comparisons of the minor allele frequencies of the four SNPs and examinations of the Hardy-Weinberg equilibrium (HWE) among ESRD patients and non-CKD ones were shown in Table 2. Both SOD2 and GPX1 SNPs satisfied the HWE in non-CKD and ESRD patients, while PPAR-*γ* Pro12Ala among ESRD cases showed deviation from HWE (*p* = 0.02). This suggests a potential association between PPAR-*γ* Pro12Ala and the presence of ESRD, as demonstrated in our previous study [11].

The genotype distributions and the other clinical characteristics were compared between the enrolled ESRD and non-CKD patients in Table 3. The mean serum creatinine (mg/dL) was significantly higher in ESRD patients (non-CKD versus ESRD, 1.0 ± 0.3 versus 11.2 ± 2.5; *p* < 0.001). In addition, the proportion of patients with DM was significantly higher in ESRD patients (non-CKD versus ESRD, 10.6% versus 37.7%; *p* < 0.001). The distributions of the SOD2 exon 2 and PPAR-*γ* exon B genotypes were significantly different between the two groups. TT genotype of SOD2 exon 2 was predominant in non-CKD patients (*p* = 0.036), while CC genotype of PPAR-*γ* exon B occurred more frequently in non-CKD patients (*p* = 0.028). There was no significant heterogeneity regarding GPX1 and PPAR-*γ* exon 6 genotypes between ESRD and non-CKD patients.

We further stratified the ESRD patients according to their ESRD etiology as DM or non-DM related, since PPAR-*γ* polymorphism is reportedly associated with development of DM nephropathy (DMN) (Table 4) [16]. About 27% patients had DMN-related ESRD. No significant difference in genotype distributions of SOD2, GPX1, and PPAR-*γ* was noted between those with or without DMN.

### 3.2. Risk of ESRD Associated with SNPs of SOD2 and GPX1

We first investigated the influence of SOD2 and GPX1 genetic polymorphism on developing ESRD. As shown in Table 5, multiple logistic regression analysis incorporating factors in Table 3 identified five significant predictors of ESRD developing among the entire cohort. Patients with DM and SOD2 exon 2 CC genotype had significantly higher risk of developing ESRD. Comorbid DM played an important role in determining the influence on ESRD risk brought by SOD2 and GPX1 genotypes. It is interesting to observe that patients without DM but carrying SOD2 exon 2 TT genotypes had lower chances of developing ESRD (OR 0.699, 95% confidence interval [CI] 0.52–0.94; *p* = 0.018).

### 3.3. Risk of ESRD Associated with SNPs of SOD2, GPX1, PPAR-*γ* Pro12Ala, and C161T

We further combined the previously reported PPAR-*γ* SNPs (i.e., Pro12Ala and C161T) and SOD2 as well as GPX1 SNPs in statistical models for a more in-depth analysis. Regression analysis identified multiple significant predictors of developing ESRD (Table 6). Individuals without DM but with SOD2 exon 2 TT genotype were still at lower risk of ESRD (OR 0.69, 95% CI 0.51–0.93; *p* = 0.014). Additionally, we discovered that PPAR-*γ* SNPs and GPX1 SNPs interacted with regard to their effect on ESRD risk. Patients with PPAR-*γ* exon 6 TT genotype and exon B GG genotype displayed a high risk of ESRD (*p* < 0.001), while exon 6 CC genotype and exon B CC genotype were protective against ESRD (OR 0.78, 95% CI 0.61–0.99; *p* = 0.04). Patients with simultaneous GPX1 exon 2 CC genotype and PPAR-*γ* exon B GG genotype had significantly lower ESRD risk (*p* < 0.001).

## 4. Discussion

In the current study, we addressed the issue whether antioxidative enzymes (SOD2 and GPX1) SNPs affect the risk of developing ESRD, in a large cohort of patients of Han origin, and their potential interactions with other known ESRD risk-modifying SNPs (PPAR-*γ*). We discovered that risk conferred by SOD2 and PPAR-*γ* SNPs in Han Chinese population is modified in the presence of DM, and interactions exist among different SNPs. Particularly, PPAR-*γ* exon B GG genotypes increased risk of ESRD in patients without DM and those with exon 6 TT genotype. In addition, the influence of SOD2 SNPs on the risk of ESRD differed between patients with and without DM. These findings suggested a renewed explanation for the associations between both antioxidants, PPAR-*γ* SNPs and DM, non-DM related ESRD in the literature.

The SOD2 SNPs, rs4880, substitute a C to T at position 2734 and change amino acid coding from alanine to valine at position 16 (Ala16Val). This alteration leads to mRNA instability and reduced SOD2 expression. SOD2 Ala16Val SNPs have been found to correlate variably with different cancer susceptibility. A meta-analysis concluded that Ala allele conferred a borderline elevated risk of breast cancer [17]. Other researchers identified a higher risk of prostate cancer (odds ratio, 1.16) and increased aggressiveness among Ala alleles of SOD2 exon 2 SNP carriers [18]. Similarly, carriers of SOD2 Ala16Val exhibited a higher risk of bladder cancer, particularly among concurrent smokers [19]. On the other hand, SOD2 Ala16Val SNPs might also play a role in the susceptibility of multiple metabolic disorders. Ala allele has been linked with elevated risk of diabetic macular edema and diabetic retinopathy in Asian population [20, 21]. Moreover, SOD2 Ala16Val SNPs also serve as an important risk factor for atherosclerotic burden, cardiac events, and nonalcoholic steatohepatitis [22–24]. These vascular and metabolic abnormalities among SOD2 Ala16Val carriers, coupling with the susceptibility to urologic malignancies, might pave the way for incident CKD development, progression, and ESRD [25]. Indeed, subsequent study revealed that Val allele of SOD2 exon 2 carriers had a 3-4-fold higher risk of rapid renal function decline compared to the others, but this was established only in Caucasians [10]. Based upon a large cohort of Han Chinese patients, we discovered that CC genotype of SOD2 exon 2 portended significantly higher risk of ESRD among those with DM, while TT genotype decreased the risk among non-DM ones (Tables 5 and 6). Although our identified at-risk allele (Ala) in Han Chinese population differs from that of Caucasians (Val) in most studies, both findings underline the importance of SOD2 exon 2 SNPs in modifying the risk of CKD progression as well as ESRD.

GPX1, a selenoenzyme that catalyzes the breakdown of various peroxides into O_2_ and H_2_O, is produced by nearly all tissues and serves as a cellular barrier against oxidative stress. Past studies focusing on the role of GPX1 SNPs mostly included GPX1 SNP rs1050450, which causes a C to T mutation [18]. GPX1 SNP rs1050450 is found to influence the risk of lung cancer and bladder cancer [26]. Risk of vascular calcification and atherosclerosis is also affected by Leu allele of GPX1 SNPs [24, 27]. However, available evidence did not agree that GPX1 SNP rs1050450 plays a significant role in CKD progression or renal allograft dysfunction [10, 28]. We similarly did not detect significant influences of GPX1 Pro197Leu SNPs on the risk of ESRD in Han Chinese population (Tables 5 and 6). This is compatible with the literature and further supports the finding that GPX1 SNPs alone did not affect the risk of ESRD in most reports. However, a subgroup of patients with GPX1 CC genotype did demonstrate lower risk of ESRD, that is, those with concurrent PPAR-*γ* exon B GG (Table 6). A previously undetected interaction between antioxidants SNPs and PPAR-*γ* SNPs might exist and warrants further investigation.

The interactions between DM/antioxidant SNPs and within different antioxidant SNPs on the risk of ESRD are interesting. From our findings, we propose that DM and CC genotype might act synergistically to elevate the risk of subsequent ESRD or that CC genotype might promote the development of diabetic nephropathy (Table 5). However, past studies failed to disclose any significant effect of SOD2 Ala16Val SNPs on the presence of DM, but, on the contrary, TT genotype carrier of SOD2 exon 2 was more likely to be found in Caucasian and Japanese patients with DM nephropathy than those without [8, 20, 29]. It is likely that SOD2 exon 2 SNPs, specifically CC genotypes, contribute to the risk of DM nephropathy among Han Chinese population. Furthermore, we also discovered that patients without DM were at significantly lower risk of ESRD if they carried TT genotypes of SOD2 exon 2 (Tables 5 and 6). This could be a circumstantial evidence supporting the detrimental effect of CC genotypes on ESRD, while it alternatively could suggest a protective effect brought by TT genotypes against non-DM ESRD. Finally, our findings that PPAR-*γ* Pro12Ala interacted with non-DM status on the effect of risk for ESRD could also be a token of gene-environmental interaction, as PPAR-*γ* Pro12Ala has been found to act together with body mass index or dietary fatty acid intake to increase cardiometabolic risk [30, 31].

Several lines of evidence suggested that interactions between different SNPs could modify the risk of CKD and its progression. FIND (Family Investigation of Nephropathy and Diabetes) study group found that *α*-actinin-4 polymorphism could interact with APOL1 SNPs with regard to the risk of non-DM related ESRD in African Americans [32]. The same group subsequently utilized a large cohort study to identify podocin (NPHS2) and APOL1 SNPs might jointly promote nondiabetic nephropathy in African Americans, although the mechanisms of synergism still remained undetermined [33]. Importantly, PPAR-*γ* activation has been reported to regulate renal mesangial inflammation and modulate renal fibrosis through p65 and NF-*κ*B pathways [34]. Furthermore, experimental evidence also suggested that PPAR-*γ* participates in regulating oxidative stress and acute kidney injury [35]. Consequently, we could expect that PPAR-*γ* SNPs and even antioxidant SNPs might interact with regard to their influence on the risk of ESRD. In this study, we discovered that PPAR-*γ* SNPs, Pro12Ala, C161T, and GPX1 SNPs might interact with each other, lending support to our hypothesis that there could be interplay between different SNPs concerning their clinical effects.

Our study has its strength and limitations. The case number was large, and our investigation on the reciprocal relationship between different SNPs among a population receiving less attention, Han Chinese, made this study more appealing. However, the current study was also limited by its clinical setting and the limited inclusion of antioxidant SNPs. Further research utilizing more comprehensive methods for SNPs survey might be needed in the future.

## 5. Conclusion

Using a large Han Chinese-based cohort, we studied the modifying effect of SOD2, GPX1, and PPAR-*γ* SNPs on the risk of ESRD. We found that CC genotype of SOD2 elevated the risk of ESRD in patients with DM, while TT genotype of SOD2 decreased the risk in patients without DM. In addition, interactions between PPAR-*γ* exon B/exon 6 and PPAR-*γ* exon B/GPX1 exon 2 as ESRD risk determinants were also detected. These findings pave the way for a better understanding of the genetic influences on the susceptibility to ESRD among the Han Chinese, a population with high ESRD prevalence.

## Figures and Tables

**Table 1:** Primers and restriction enzymes for PCR-RFLP.

Gene/SNP	Primers for PCR	Restriction enzyme	Size of digestion products (base pairs)
SOD2/V16A	5′-CTGACCGGGCTGTGCTTTCT-3′	BsaWI	TT: ~50, 175
	5′-CAACGCCTCCTGGTACTTCT-3′		CT: ~50, 175, 225
			CC: ~225

GPX1/P197L	5′-GCTTCCAGACCATTGACATC-3′	ApaI	CC: ~53, 261
	5′-TCCCAAATGACAATGACACAG-3′		CT: ~53, 261, 314
			TT: ~314

PPAR-*γ*/P12A	5′-GCCAATTCAAGCCCAGTC-3′	BstUI	CC: ~270
	5′-GATATGTTTGCAGACAGTGTATC		CG: ~43, 227, 270
	AGTGAAGGAATCGCTTTCCG-3′		GG: ~43, 227

PPAR-*γ*/C161T	5′-TTTGACTGAACCCCCTGTTG-3′	NlaIII	CC: ~101, 245
	5′-CAGAATAGTGCAACTGGAAGA-3′		CT: ~41, 101, 204, 245
			TT: ~41, 101, 204

**Table 2:** Comparisons of the minor allele frequencies of the four SNPs and examinations of the Hardy-Weinberg equilibrium among the entire cohort.

SNPs	rs #	Nucleotide Change (amino acid change)	Location	Controls (*n* = 780)		Cases (*n* = 671)	
				MAF	HWE *p* value	MAF	HWE *p* value
SOD2	rs4880	T → C (Val → Ala)	exon 2	13.97%	0.768	17.36%	0.423
GPX1	rs1050450	C → T (Pro → Leu)	exon 2	5.38%	0.720	5.29%	0.249
P12A	rs1801282	C → G (Pro → Ala)	exon B	3.08%	0.526	4.99%	0.020
C161T	rs3856806	C → T (His → His)	exon 6	24.29%	0.697	26.53%	0.488

HWE = Hardy-Weinberg equilibrium.

MAF = minor allele frequency.

**Table 3:** Characteristics of patients with ESRD and those without CKD.

Variables	Non-CKD	ESRD patients	*p* ^*∗*^
Number of subjects	780	671	
Age (years)	60.0 ± 19.2	58.9 ± 14.6	<0.001
Male	410 (52.6%)	327 (48.7%)	0.155
DM	83 (10.6%)	253 (37.7%)	<0.001
Serum creatinine (mg/dL)	1.0 ± 0.3	11.2 ± 2.5	<0.001
SOD2 exon 2 genotype			0.036
TT	576 (73.85%)	455 (67.8%)	
CT	190 (24.36%)	199 (29.66%)	
CC	14 (1.79%)	17 (2.53%)	
GPX1 exon 2 genotype			1.000
CC	697 (89.36%)	600 (89.42%)	
CT	82 (10.51%)	71 (10.58%)	
TT	1 (0.13%)	0 (0.00%)	
PPAR-*γ* exon B genotype			0.028
CC	733 (93.90%)	609 (90.99%)	
CG	46 (5.97%)	57 (8.30%)	
GG	1 (0.13%)	5 (0.72%)	
PPAR-*γ* exon 6 genotype			0.382
CC	449 (57.69%)	366 (55.08%)	
CT	283 (36.21%)	254 (37.63%)	
TT	48 (6.10%)	51 (7.30%)	

^*∗*^Continuous variables were tested by Kruskal-Wallis test, whereas categorical variables were tested by Fisher's exact test.

**Table 4:** Characteristics of ESRD patients due to DMN and non-DMN related ESRD.

Variables	Non-DMN-related ESRD patients	DMN-related ESRD patients	*p* ^*∗*^
Number of subjects	492	179	
SOD2 exon 2 genotype			0.716
TT	330 (67.1%)	125 (69.8%)	
CT	150 (30.5%)	49 (27.4%)	
CC	12 (2.4%)	5 (2.8%)	
GPX1 exon 2 genotype			0.258
CC	444 (90.2%)	156 (87.2%)	
TC	48 (9.8%)	23 (12.8%)	
TT	0 (0.00%)	0 (0.00%)	
PPAR-*γ* exon B genotype			0.284
CC	451 (91.7%)	158 (88.3%)	
CG	37 (7.5%)	20 (11.2%)	
GG	4 (0.8%)	1 (0.6%)	
PPAR-*γ* exon 6 genotype			0.454
CC	263 (53.5%)	103 (57.5%)	
CT	193 (39.2%)	61 (34.1%)	
TT	36 (7.3%)	51 (8.4%)	

^*∗*^Continuous variables were tested by Kruskal-Wallis test, whereas categorical variables were tested by Fisher's exact test.

**Table 5:** Multivariate analysis^*∗*^ of the predictors of dialysis by fitting multiple logistic regression model with the stepwise variable selection method, including SOD2 and GPX1 polymorphisms^#^.

Covariates	Regression coefficient	Standard error	*t* value	*p* value	Odds ratio	95% confidence interval	
Intercept	−0.780	0.150	−5.194	<0.001	—	—	—

Age (between 28 and 65)	1.578	0.124	12.724	<0.001	4.847	3.801	6.180
Male	−0.252	0.123	−2.056	0.040	0.777	0.611	0.988
DM	1.757	0.185	9.520	<0.001	5.792	4.035	8.316

Non-DM × SOD2 exon 2 TT	−0.358	0.151	−2.378	0.018	0.699	0.520	0.939

^*∗*^Multiple logistic regression model: *n* = 1451, adjusted generalized *R* ^2^ = 0.296, estimated area under the receiver operating characteristic (ROC) curve = 0.762, and the Hosmer and Lemeshow goodness-of-fit chi-square test *p* = 0.003 (df = 8).

^#^Variables with odds ratio (OR) of extreme values were listed below: DM × SOD2 exon 2 CC, OR 3.6 × 10^5^ (*p* < 0.001).

**Table 6:** Multivariate analysis^*∗*^ of the predictors of dialysis by fitting multiple logistic regression model with the stepwise variable selection method, including SOD2, GPX1, and PPAR-*γ* polymorphisms^#^.

Covariates	Regression coefficient	Standard error	*t* value	*p* value	Odds ratio	95% confidence interval	
Intercept	−0.655	0.162	−4.038	<0.001	—	—	—

Age (between 28 and 65)	1.585	0.125	12.689	<0.001	4.879	3.820	6.232
Male	−0.240	0.123	−1.953	0.051	0.786	0.618	1.001
DM	1.785	0.186	9.617	<0.001	5.957	4.141	8.569

Non-DM × SOD2 exon 2 TT	−0.370	0.151	−2.452	0.014	0.691	0.514	0.928

PPAR-*γ* exon 6 CC × PPAR-*γ* exon B CC	−0.254	0.123	−2.059	0.040	0.778	0.609	0.988

^*∗*^Multiple logistic regression model: *n* = 1451, adjusted generalized *R* ^2^ = 0.304, estimated area under the receiver operating characteristic (ROC) curve = 0.769, and the Hosmer and Lemeshow goodness-of-fit chi-square test *p* = 0.0047 (df = 8).

^#^Variables with odds ratio (OR) of extreme values were listed below: DM × SOD2 exon 2 CC, OR 2.7 × 10^6^ (*p* < 0.001); non-DM × PPAR-*γ* exon B GG, OR 3.8 × 10^13^ (*p* < 0.001); PPAR-*γ* exon 6 TT × PPAR-*γ* exon B GG, OR 8.2 × 10^11^ (*p* < 0.001); GPX1 exon 2 CC × PPAR-*γ* exon B GG, OR 1.9 × 10^−7^ (*p* < 0.001).
